# Alcohol Use Disorder Medication Coverage and Utilization Management in Medicaid Managed Care Plans

**DOI:** 10.1001/jamanetworkopen.2025.0695

**Published:** 2025-03-13

**Authors:** Maureen T. Stewart, Sage R. Feltus, Christina M. Andrews, Andrea Acevedo, Cindy Parks Thomas, Jeffrey Bratberg, Constance M. Horgan, Dominic Hodgkin, Rachel Sayko Adams

**Affiliations:** 1 Institute for Behavioral Health, The Heller School for Social Policy and Management, Brandeis University, Waltham, Massachusetts; 2 Department of Health Law, Policy and Management, Boston University School of Public Health, Boston, Massachusetts; 3 Department of Health Services Policy and Management, Arnold School of Public Health, University of South Carolina, Columbia; 4 Booz Allen Hamilton Inc, McLean, Virginia; 5 Department of Pharmacy Practice and Clinical Research, University of Rhode Island College of Pharmacy, Kingston

## Abstract

**Importance:**

Evidence-based, patient-centered treatment for alcohol use disorder (AUD) can include pharmacotherapy with naltrexone, acamprosate, or disulfiram; however, these medications are rarely used. Medicaid managed care plans (MCPs) manage health services for nearly 80% of Medicaid enrollees and are the largest payer for addiction treatment services. Little is known about Medicaid MCP policies for AUD medications.

**Objectives:**

To describe Medicaid MCPs’ coverage and management of acamprosate, naltrexone, and disulfiram for AUD and examine associations of plan characteristics and state policies with medication coverage.

**Design, Setting, and Participants:**

In this cross-sectional study, a content analysis was performed of 2021 insurance benefit data for 241 comprehensive Medicaid MCPs in states using Medicaid managed care, as well as secondary sources. Data were analyzed from May to August 2024.

**Main Outcomes and Measures:**

Medicaid MCP-reported medication coverage and utilization management requirements (eg, prior authorization, quantity limit requirements) for acamprosate, disulfiram, and oral and injectable naltrexone together and for each medication separately. Independent variables included plan characteristics (profit status, market share) and the state policy environment in which plans are embedded (Section 1115 substance use disorder waiver, state-defined preferred drug list). Regressions examined associations of plan characteristics and state policies with medication coverage.

**Results:**

In this cross-sectional content analysis of 241 comprehensive Medicaid MCPs in 2021, 217 (90.0%) covered at least 1 medication for AUD: 132 (54.7%) covered acamprosate, 203 (84.2%) covered oral naltrexone, 175 (72.6%) covered injectable naltrexone, 152 (63.0%) covered disulfiram, and 103 (42.7%) covered all 4 medications. Prior authorization and quantity limits were rarely applied, except for injectable naltrexone, for which 75 plans (42.8%) imposed at least 1 of these utilization management requirements.

**Conclusions and Relevance:**

This study suggests that efforts to expand AUD medication prescribing may be limited by gaps in health insurance coverage. Medicaid MCPs and states can support AUD medication utilization by covering these medications without applying utilization management strategies.

## Key Points

**Question:**

How do Medicaid managed care plans (MCPs) cover and manage medications for alcohol use disorder (AUD)?

**Findings:**

In this cross-sectional study, a content analysis of publicly available data from all 241 comprehensive Medicaid MCPs in 2021 revealed that 103 plans (42.7%) covered all approved medications (acamprosate, naltrexone, and disulfiram) for AUD. Prior authorization and quantity limits were used rarely, except for injectable naltrexone.

**Meaning:**

This study suggests that expanding medication use for AUD and providing patient-centered care may be undermined by insurance coverage limitations.

## Teaser

This cross-sectional study describes Medicaid managed care plans’ coverage and management of acamprosate, naltrexone, and disulfiram for alcohol use disorder and examines associations of plan characteristics and state policies with medication coverage.

## Introduction

Alcohol use disorder (AUD) is an underrecognized, significant public health issue with adverse effects on individuals and society.^1^ An estimated 28.1 million adults in the US have AUD,^2^ which is associated with substantial morbidity, including increased rates of cancer, heart disease, diabetes, and injuries.^3,4^ Alcohol-related deaths increased 70% from 2012 to 2022, with sharp increases observed during the COVID-19 pandemic.^5^ More than 178 000 individuals in the US died in 2020 and 2021 from excessive alcohol use, a 23% increase from 2016 and 2017.^6^ An estimated 1 in 5 deaths among people aged 20 to 49 years are attributable to alcohol.^4^ Furthermore, alcohol-related morbidity and mortality are increasing rapidly among Black and Hispanic individuals,^7^ women,^8,9^ and people living in rural areas of the US.^10^

Evidence-based treatments for AUD include behavioral therapies and medications.^11,12^ The 4 US Food and Drug Administration (FDA)–approved medications for AUD (MAUD), acamprosate, disulfiram, and naltrexone (oral and extended-release injectable), are safe, relatively inexpensive, and cost-effective.^13,14^ Oral naltrexone and acamprosate have the strongest evidence for AUD treatment and are recommended by the American Psychiatric Association as first-line treatments for moderate to severe AUD.^15^ Naltrexone may help with reducing heavy drinking and craving^16^ and has been associated with reduced progression of alcohol-related liver disease.^17^ Acamprosate is associated with maintenance of abstinence from alcohol.^18,19^ Disulfiram may be less effective than other MAUD but can help decrease the likelihood of relapse among individuals adherent to the medication regimen; American Psychiatric Association clinical guidelines recommend disulfiram for patients with a goal of abstinence from alcohol.^15,20,21^ MAUD regimens differ based on route of administration (oral or injectable), dosing schedules,^22^ contraindications,^23^ adverse effect profiles,^11^ and the individual’s ability to initiate medication while still drinking alcohol.^12^ Given these differences, true patient-centered care requires access to the range of medications.^11,15^ These medications are relatively low cost (<$50 for a 1-month supply),^24,25,26^ with the exception of injectable naltrexone, which is not available as generic (>$1000 per monthly injection).^27^ Despite clinical guideline recommendations, among the estimated 28.1 million adults identified in a community sample as meeting criteria for having AUD, less than 3% received MAUD in 2023 and less than 2% of Black and Hispanic individuals with AUD received MAUD.^2^

Medicaid has the potential to be a critical policy lever to expand access to MAUD for Medicaid enrollees with AUD. Most state Medicaid programs contract with managed care plans (MCPs) to deliver and manage health services, including substance use disorder (SUD) treatment.^28,29^ Approximately 75% of the 80 million Medicaid enrollees are in Medicaid MCPs.^30^ Although the use of MAUD is higher among the Medicaid population (5.1%) than for those privately insured (1.0%) and uninsured (1.3%),^2^ there is a need to improve treatment use for Medicaid MCP enrollees.^31^ Little is known about how Medicaid MCPs cover and manage MAUD.

State policies may mandate Medicaid MCP coverage or require utilization management of MAUD.^32^ However, within the broad constraints of federal and state policies, MCPs have considerable discretion to develop medication coverage policies and, for covered medications, to define utilization management requirements, such as prior authorization and quantity limits, that may influence access to MAUD. Prior authorization helps ensure appropriate use of care and encourage less-expensive comparable treatments.^33^ However, prior authorization requirements for psychiatric medications have been associated with increased utilization of emergency services, higher overall medical costs, and mortality.^34,35^ Quantity limits are used by insurance companies as a mechanism to reduce overutilization but can also affect practitioners’ ability to tailor treatment to individuals’ needs and stage of treatment.^36^ Quantity limits may cap the number of days filled at a time or the daily dosage allowed before additional authorization from the MCP is required.

Although coverage and utilization management of MAUD in Medicaid MCPs are unknown, studies^37,38^ suggest they may vary by MCP characteristics and state policies. For-profit ownership has been associated with poorer health care access in Medicaid managed care.^37,38^ Managed care plans may manage and be at risk for substance use treatment costs, or they may contract with a managed behavioral health organization (“carve out”) that specializes in such services^39^; these arrangements may have implications for access to care.^40^ Furthermore, state policies such as state Section 1115 SUD waivers are designed to expand access to SUD treatment.^41^ Inclusion of specific medications on a state-defined uniform preferred drug list (PDL) is associated with more dispensing of those medications.^42^

National efforts are under way to expand MAUD as 1 strategy to address the gaps in AUD treatment utlization.^43,44,45^ Information about Medicaid MCP coverage and utilization management policies for MAUD and the factors that may be associated with benefit design choices is essential to inform these strategies. The present study characterizes MAUD coverage and utilization management requirements in Medicaid MCPs. We also examine associations of Medicaid MCP characteristics and the state-policy environment with MAUD coverage policies. Identifying the potential structural barriers to expanding MAUD use and the factors associated with MCP MAUD policies will strengthen efforts to improve MAUD treatment use and help ensure equitable access to MAUD.

## Methods

### Study Design and Data Sources

For this cross-sectional study, we used the Centers for Medicaid & Medicare Services (CMS) website^46^ to identify all comprehensive Medicaid MCPs across 39 states and the District of Columbia in 2021. Plans that provided coverage only for specific populations were excluded (eg, plans only for people with serious mental illness). A total of 241 Medicaid MCPs met study inclusion criteria (eTable 1 in Supplement 1). The Brandeis University institutional review board determined that this study did not qualify as human participants research and therefore was exempt from review. Study reporting followed the Strengthening the Reporting of Observational Studies in Epidemiology (STROBE) reporting guideline.

We conducted a content analysis of publicly available 2021 insurance benefit documentation from these 241 plans. A search protocol was used to review plan PDLs (for medication coverage and utilization management policies) and member handbooks (for behavioral health contracting arrangements). Plan profit status was collected from MCP websites and Internal Revenue Service documents. We obtained National Committee for Quality Assurance (NCQA) accreditation for each MCP from the NCQA website.^47^ Plan enrollment size was obtained from the CMS enrollment report^48^ to calculate market share. We gathered documentation of Section 1115 SUD waivers from the CMS^49^ and state AUD prevalence rates from the National Survey on Drug Use and Health.^50^

### Measures

#### Outcome Variables: Coverage and Utilization Management of MAUD

Acamprosate, disulfiram, and injectable and oral naltrexone were each coded as covered if the medication was listed on the MCP’s PDL. For each covered medication, we coded prior authorization and quantity limit requirements listed in the PDL. If prior authorization or quantity limit requirements were not listed, we coded this as unspecified. We created a variable that identified plans that covered all MAUD.

#### Independent Variables

##### MCP Characteristics

Managed care plan characteristics included plan profit status (for profit or nonprofit), behavioral health contracting arrangement (managed internally by the MCP or carved out to the state or managed behavioral health organization), and NCQA accreditation (yes or no). We calculated MCP market share, defined as each MCP’s share of the state Medicaid managed care enrollment, and dichotomized the variable at the 75th percentile (<24.9% market share = small; ≥24.9% market share = large).

##### State Policy Environment

State policy environment variables included the following characteristics in 2021: obtained a Section 1115 SUD waiver (yes or no) and used a state-defined uniform PDL (yes or no). We included an indicator of past-year AUD prevalence in the state among adults aged 18 years or older, dichotomized at the 75th percentile (<12.1% = low; ≥12.1% = high) (eTable 2 in Supplement 1).

### Statistical Analysis

Statistical analysis was performed from May to August 2024. We characterized the sample by generating descriptive statistics for the number and percentage of MCPs and enrollees by plan characteristics and state policy environment. We calculated descriptive and bivariate statistics for the number and percentage of MCPs and enrollees by coverage and utilization management policies for all MAUD and for each medication separately and by plan characteristics and state environment. We mapped the percentage of MCP plans covering all MAUD by state and the percentage of enrollees in plans covering all MAUD by state.

We estimated 5 random-effects multivariate logistic regression models, using coverage of all MAUD and each of the 4 medications separately as dependent variables with plan characteristics and state policy indicators as explanatory variables, controlling for state AUD prevalence. Explanatory variables were not correlated and were omitted when the cell size was less than 5 observations. Data analysis was performed using Stata, version 18.0 (StataCorp LLC). Multivariable analyses of utilization management policies were not conducted due to small sample size and multicollinearity. All *P* values were from 2-sided tests and results were deemed statistically significant at *P* < .05.

## Results

The Table describes the characteristics of MCPs. Of the 241 Medicaid MCPs managing care for approximately 58 million enrollees in 2021, plans were almost evenly split between nonprofit and for profit, and most plans managed behavioral health internally, were accredited by the NCQA, and had a market share less than the 75th percentile. Most MCPs (insuring approximately 38 million enrollees) operated in states that had Section 1115 SUD waivers and did not have a state-defined uniform PDL.

**Table.** Medicaid MCP Characteristics and State Policies in 2021

MCP characteristic	No. (%)	
	MCPs (N = 241)	Medicaid enrollees (N = 57 534 431)
Profit status		
For profit	117 (48.6)	32 446 354 (56.4)
Nonprofit	124 (51.4)	25 088 077 (43.6)
Behavioral health contracting arrangement		
Managed internally by MCP	170 (70.5)	43 244 420 (75.2)
Carved out to other entity	71 (29.5)	14 290 011 (24.8)
NCQA accreditation status		
Accredited	154 (63.9)	40 321 641 (70.1)
Not accredited	87 (36.1)	17 212 790 (29.9)
Market share^a^		
Small	180 (74.7)	38 690 125 (67.2)
Large	61 (25.3)	18 844 306 (32.8)
State policies		
Section 1115 SUD waiver		
Had waiver	170 (70.5)	37 733 290 (65.6)
No waiver	71 (29.5)	19 801 141 (34.4)
State-defined uniform preferred drug list		
Yes	114 (47.3)	26 023 432 (45.2)
No	127 (52.7)	31 510 999 (54.8)
State alcohol use disorder prevalence, 2021^b^		
Low	158 (65.6)	40 975 799 (71.2)
High	83 (34.4)	16 558 632 (28.8)

Abbreviations: MCP, managed care plan; NCQA, National Committee for Quality Assurance; SUD, substance use disorder.

^a^ Small market share refers to market share less than the 75th percentile (<24.9% market share) and large market share refers to market share of the 75th percentile or greater (≥24.9% market share).

^b^ Low alcohol use disorder prevalence rate refers to less than the 75th percentile (<12.1%) and high alcohol use disorder prevalence rate refers to the 75th percentile or greater (≥12.1%).

### MAUD Coverage and Utilization Management Requirements

**Figure 1.**
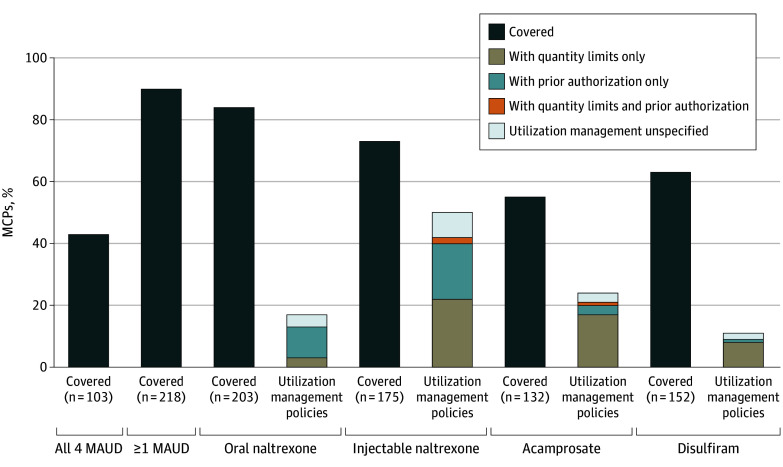
Alcohol Medication Coverage and Utilization Management Policies in Medicaid Managed Care Plans (MCPs), 2021 (N = 241) Utilization management policies are considered only among plans that have coverage for the medication. MAUD indicates medications for alcohol use disorder.

Coverage and utilization management requirements by medication are shown in Figure 1. Of the 241 MCPs, 217 (90.0%) covered at least 1 medication for AUD; 103 MCPs (42.7%) covered all 4 AUD medications. A total of 203 MCPs (84.2%) covered oral naltrexone; of these, 169 (83.3%) did not require prior authorization or quantity limits. Injectable naltrexone was covered by 175 MCPs (72.6%); 87 (49.7%) did not require prior authorization or quantity limits. Acamprosate was covered by 132 MCPs (54.7%); 100 (75.8%) did not require prior authorization or quantity limits. A total of 152 MCPs (63.0%) covered disulfiram; 135 (88.9%) did not require prior authorization or quantity limits. Prior authorization and quantity limits were rarely used for oral naltrexone and disulfiram, and acamprosate, but were used for injectable naltrexone in 75 MCPs (42.8%). Some MCPs were missing utilization management information for oral naltrexone (n = 8), injectable naltrexone (n = 13), acamprosate (n = 4), and disulfiram (n = 4).

**Figure 2.**
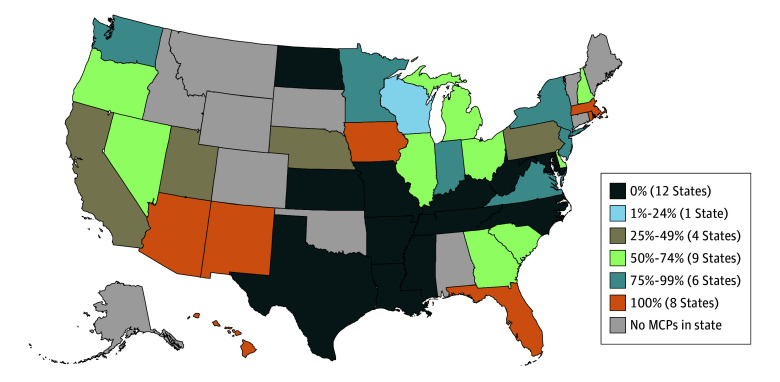
Percentage of Comprehensive Medicaid Managed Care Plans (MCPs) Covering All Medications for Alcohol Use Disorder by State, 2021

**Figure 3.**
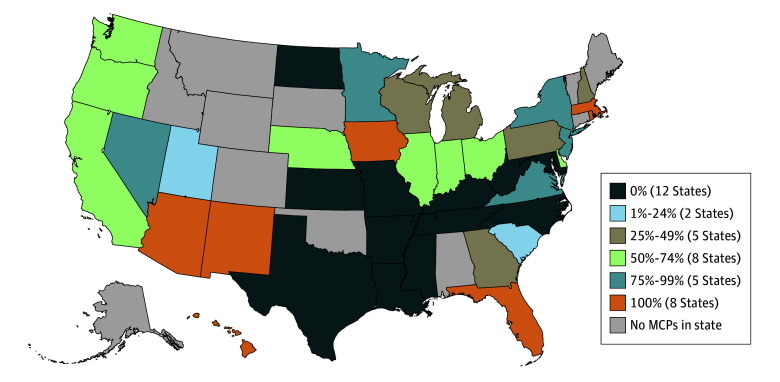
Percentage of Enrollees in Medicaid Managed Care Plans (MCPs) Covering All Medications for Alcohol Use Disorder by State, 2021 (N = 57 534 431)

Twenty-eight states (70.0%) had at least 1 MCP covering all 4 MAUD, while in 12 states (30.0%), no MCPs covered all 4 MAUD. In 8 states (20.0%), all MCPs covered all MAUD (Figure 2). In 19 states (47.5%), less than 50% of enrollees were in plans that covered all MAUD (Figure 3).

### Associations of MCP Characteristics and State Policies With MAUD Coverage

**Figure 4.**
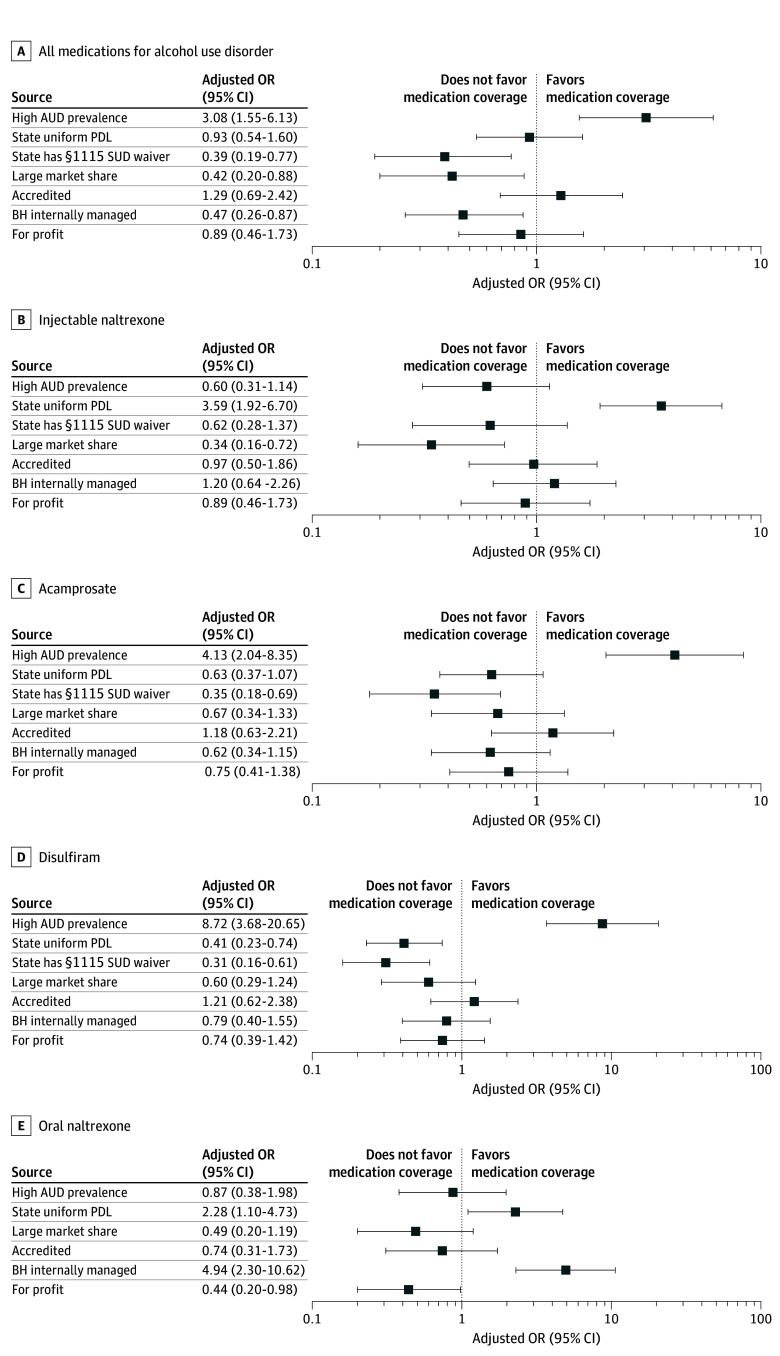
Associations of Plan Characteristics and State Policies With Coverage of Medications for Alcohol Use Disorder (MAUD) From Logistic Regression Analyses A, All MAUD. B, Injectable naltrexone. C, Acamprosate. D, Disulfiram. E, Oral naltrexone. Squares indicate adjusted odds ratios (ORs) and horizontal lines represent the 95% CIs of the estimate. When an OR and corresponding 95% CI do not pass the vertical line representing 1, that variable was statistically significant in the regression model. The scale for the panel showing associations with disulfiram and oral naltrexone coverage is different from the other panels’ scales (0.1-100 instead of 0.1-10). The x-axis is plotted in a log scale. AUD indicates alcohol use disorder; BH, behavioral health; PDL, preferred drug list; and SUD, substance use disorder.

Managed care plans that managed behavioral health internally were less likely to cover all MAUD (odds ratio [OR], 0.47 [95% CI, 0.26-0.87]; *P* = .02) when controlling for other variables (Figure 4). Plans operating in states with Section 1115 SUD waivers were also less likely to cover all MAUD (OR, 0.39 [95% CI, 0.19-0.77]; *P* = .007). Managed care plan characteristics and state policies were also associated with coverage of specific medications. For-profit MCPs were less likely to cover oral naltrexone (OR, 0.44 [95% CI, 0.20-0.98]; *P* = .05). Managed care plans that managed behavioral health internally were more likely to cover oral naltrexone (OR, 4.94 [95% CI, 2.30-10.62]; *P* < .001), as were MCPs in states with a state-defined uniform PDL (OR, 2.28 [95% CI, 1.10-4.73]; *P* = .003). Managed care plans in states with a state-defined uniform PDL were also more likely to cover injectable naltrexone (OR, 3.59 [95% CI, 1.92-6.70]; *P* < .001).

## Discussion

Less than half of Medicaid MCPs (103 of 241 [42.7%]) covered all 4 MAUD. Clinical considerations and shared decision-making between patients and clinicians is an important component of AUD treatment.^15^ Each MAUD has different considerations, including adverse effect profiles, route of administration, and dosing schedules, that must be evaluated by patients and their clinicians. For example, some people will not want injections or will not tolerate injection site pain, making injectable naltrexone a poor fit. Conversely, monthly injections could be optimal for people who struggle with adherence to daily medications (eg, oral naltrexone or acamprosate dosing 3 times daily). Therefore, covering all MAUD may support enrollees seeking MAUD to choose the medication that works best for them. Future work should examine how MCP policies are associated with enrollees’ MAUD use.

We observed variation in the coverage of the different MAUD. Oral and injectable naltrexone were more commonly covered than other MAUD. The 2018 Substance Use-Disorder Prevention that Promotes Opioid Recovery and Treatment for Patients and Communities (SUPPORT) Act^51^ requires Medicaid plans to cover all FDA-approved medications for opioid use disorder, including injectable naltrexone. This requirement may help explain the higher coverage for injectable naltrexone even though it is the most expensive medication. This finding suggests that requiring medication coverage is 1 pathway to address coverage gaps. Although full coverage may be optimal public health policy, given that enrollees lose coverage and then re-enroll in Medicaid, it may not be rational for MCPs because enrollees may lose eligibility before MCPs can incur longer-term cost savings from treating AUD. For states, covering all MAUD without restriction may limit Medicaid programs’ ability to negotiate rebates from pharmaceutical companies and increase state costs.^52^

Prior authorization and quantity limits were rarely used for oral naltrexone and disulfiram but were used for injectable naltrexone in 75 MCPs (42.8%). More frequent use of prior authorization and quantity limits on injectable naltrexone may be explained partially by the higher cost of the medication compared with other MAUD. Injectable formulations also require administration by a health care professional; thus, their use inherently imposes additional costs on the payer due to additional health care visits. Among MCPs that covered acamprosate, at least 20% required either prior authorization and/or quantity limits. This finding is notable given that acamprosate may be the most appropriate MAUD for individuals receiving buprenorphine or methadone maintenance for opioid use disorder who do not aim to abstain from drinking completely.^53^ This gap in coverage and the greater restrictions may disproportionately affect enrollees managing co-occurring opioid use disorder and AUD with pharmacotherapy.

The health consequences of excessive alcohol use, such as alcohol-related liver disease, cirrhosis of the liver, and alcohol-related mortality, are worse for Hispanic and Black individuals compared with their White counterparts,^54,55,56,57^ for individuals with lower socioeconomic status compared with those with higher socioeconomic status,^58,59^ and for residents in rural areas.^10^ Managed care plans that did not cover all MAUD were somewhat concentrated in states with high populations of Black residents (Mississippi and Tennessee)^60^ and Hispanic residents (Texas),^60^ in states with some of the highest percentages of residents with income below the federal poverty level (Arkansas and Louisiana),^61^ and in states with a higher proportion of residents living in rural areas (Mississippi, West Virginia, and Arkansas).^62^ Women experience greater risk for alcohol-related health consequences than men^63^ and are overrepresented in Medicaid.^64^ Managed care plans that did not cover all MAUD were in states with higher percentages of female Medicaid enrollees (Mississippi, Texas, and North Carolina) than other states.^65^ Recent research points to disparities in AUD treatment utilization within Medicaid; Latinx females in Medicaid are less likely to access treatment compared with White male counterparts.^66^ Ensuring insurance coverage for all MAUD for these populations is essential to begin to address the increased need for AUD treatment among Black and Hispanic individuals, women, individuals with low socioeconomic status, and people residing in rural areas.

This study takes a first step toward understanding how MCP characteristics and the state policy environment in which MCPs are embedded are associated with MAUD coverage. Associations identified here suggest strategies for future research and identify areas to consider targeting for policy change. Managed care plans that managed behavioral health internally were less likely to cover all MAUD, primarily because they did not cover acamprosate but were more likely to cover oral naltrexone. The association between MCP behavioral health contracting and coverage warrants further investigation. Managed care plans in states with a state-defined uniform PDL were more likely to cover oral and injectable naltrexone. Coverage for injectable naltrexone may reflect states’ efforts to comply with the 2018 SUPPORT Act.^51^ Alternative explanations may be that plans do not find evidence for acamprosate and disulfiram compelling, that enrollees and clinicians have not asked plans to include the medications on the PDL, that enrollment patterns among enrollees do not incentivize including the medications, or that plans are not adequately reimbursed for enrollees with SUDs who generally have higher health care expenditures^67,68^ and therefore do not document MAUD on the PDL; more research is needed to understand these associations.

The results of this study emerge at a key moment in addressing escalating alcohol-related morbidity and mortality in the US.^5^ In 2023, the Substance Abuse and Mental Health Services Administration launched a national Providers Clinical Support System–Medications for Alcohol Use Disorder, focused on enhancing the capacity of the health care workforce to identify and treat individuals with AUD using MAUD.^43,69^ The US Department of Health and Human Services set an agency goal of reducing emergency department visits for conditions including acute alcohol use by 10% by 2025 by increasing access and utilization of prevention, intervention, treatment, and recovery services.^44^ Prior work has shown that initiating MAUD before hospital discharge is associated with a substantial reduction in the risk of returning to the hospital or dying within 30 days.^70^ Widespread coverage of all MAUD could support these initiatives. State or federal requirements for Medicaid MCPs to clearly report MAUD coverage would complement these efforts.

### Limitations

This study has several limitations. This study was a cross-sectional analysis of coverage and utilization management of MAUD in 2021, and no causality can be inferred from the associations identified in the analyses. Our study was limited to the publicly available insurance documentation that is most likely to be accessed by enrollees and clinicians. In 13 cases, utilization management information was not reported. It is important for MCPs to clearly describe the benefits and restrictions because this information is needed by enrollees to choose plans and clinicians to deliver treatment while limiting administrative hurdles. Plans typically did not report details on quantity limits, making it challenging to assess how much of a barrier they represent; additional work to investigate these details is needed. Medicaid MCPs may pay for medications not included on the PDL, but this information would not be known to enrollees making plan decisions or clinicians making treatment decisions.

## Conclusions

In this cross-sectional study of the 241 Medicaid MCPs in 2021, fewer than half of plans covered all MAUD. Federal efforts to expand MAUD treatment may experience challenges due to coverage and utilization management policies. More research is needed to understand why MCPs do not report covering these safe, effective, and relatively inexpensive medications. States and Medicaid MCPs should examine PDLs to ensure all MAUD are included and should continue limiting utilization management. Coverage is a necessary first step to address low MAUD use rates; it will not be sufficient alone. An approach confronting stigma and patient and clinician knowledge and beliefs about MAUD^71^ is necessary to bring attention to the growing AUD public health crisis.

## Notes

Supplement 1. **eTable 1.** States Included and Excluded From the Analyses **eTable 2.** State Policy Environment Characteristics, 2021

Supplement 2.
Data Sharing Statement
