# Supplementary material for: Alcohol Use Disorder Medication Coverage and Utilization Management in Medicaid Managed Care Plans
Source: JAMA Netw Open. 2025 Mar 13;8(3):e250695. doi: 10.1001/jamanetworkopen.2025.0695 (PMC11907321; doi:10.1001/jamanetworkopen.2025.0695)
Supplement: Supplement 1. — eTable 1. States Included and Excluded From the Analyses eTable 2. State Policy Environment Characteristics, 2021 [file jamanetwopen-e250695-s001.pdf]

Supplementary Online Content

Stewart MT, Feltus SR, Andrews CM, et al. Alcohol use disorder medication coverage and utilization management in Medicaid managed care plans. *JAMA Netw Open.* 2025;8(3):e250695. doi:10.1001/jamanetworkopen.2025.0695

eTable 1. States Included and Excluded from the Analyses
eTable 2. State Policy Environment Characteristics, 2021

This supplementary material has been provided by the authors to give readers additional information about their work.

© 2025 Stewart MT et al. *JAMA Network Open.*

eTable 1. States Included and Excluded from the Analyses

**States included in the analyses (n=39 and District of Columbia)**	**States without comprehensive managed care contracts in 2021 (n=10)**
Arkansas, Arizona, California, Delaware, Florida, Georgia, Hawaii, Iowa, Illinois, Indiana, Kansas, Kentucky, Louisiana, Massachusetts, Maryland, Michigan, Minnesota, Missouri, Mississippi, North Carolina, North Dakota, Nebraska, New Hampshire, New Jersey, New Mexico, Nevada, New York, Ohio, Oregon, Pennsylvania, Rhode Island, South Carolina, Tennessee, Texas, Utah, Virginia, Washington, Wisconsin, West Virginia, District of Columbia	Alabama, Alaska, Connecticut, Idaho, Maine, Montana, Oklahoma, South Dakota, Vermont, Wyoming

Note. Colorado was not included in our analyses despite having Medicaid managed care plans (MCP) in 2021. We identified MCPs across all states using the Centers for Medicare and Medicaid website, which categorized Colorado’s two MCPs as primary care case management (PCCM) entities, therefore not eligible for inclusion in the study. We later learned the two entities were in fact MCPs, but we were unable to access their member materials to include them in the study.

© 2025 Stewart MT et al. *JAMA Network Open.*

eTable 2. State Policy Environment Characteristics, 2021

State	Approved Section 1115 Substance Use Disorder (SUD) Waiver[1]	Uniform preferred drug list (PDL)[2]	Alcohol use disorder (AUD) prevalence[3]
Arkansas	No	Yes	Low
Arizona	No	Yes	Low
California	Yes	No	High
Delaware	Yes	Yes	High
District of Columbia	Yes	No	High
Florida	No	Yes	Low
Georgia	No	No	Low
Hawaii	No	No	Low
Iowa	No	Yes	Low
Illinois	Yes	No	Low
Indiana	Yes	No	Low
Kansas	Yes	Yes	Low
Kentucky	Yes	Yes	Low
Louisiana	Yes	Yes	Low
Massachusetts	Yes	Yes	High
Maryland	Yes	No	Low
Michigan	Yes	No	Low
Minnesota	Yes	Yes	Low
Missouri	No	Yes	High
Mississippi	No	Yes	Low
North Carolina	Yes	Yes	Low
North Dakota	No	Yes	High
Nebraska	Yes	Yes	Low
New Hampshire	Yes	Yes	High
New Jersey	Yes	No	Low
New Mexico	Yes	No	High
Nevada	No	No	High
New York	No	No	Low
Ohio	Yes	Yes	Low
Oregon	Yes	No	High
Pennsylvania	Yes	Yes	Low
Rhode Island	Yes	No	High
South Carolina	No	No	Low
Tennessee	No	Yes	Low
Texas	No	Yes	Low
Utah	Yes	No	Low

© 2025 Stewart MT et al. *JAMA Network Open.*

State	Approved Section 1115 Substance Use Disorder (SUD) Waiver[1]	Uniform preferred drug list (PDL)[2]	Alcohol use disorder (AUD) prevalence[3]
Virginia	Yes	No	Low
Washington	Yes	Yes	High
Wisconsin	Yes	No	Low
West Virginia	Yes	Yes	Low

[1] Centers for Medicare & Medicaid Services. Section 1115 Demonstrations Medicaid. Accessed April 8, 2024. https://www.medicaid.gov/medicaid/section-1115-demonstrations/index.html

[2] Based on authors' analysis of state and MCO publicly available documents. California and Maryland had uniform drug lists in 2021 but those states allow MCOs to be more restrictive than the state PDL therefore they were marked as ‘no’

[3]Substance Abuse and Mental Health Services Administration. 2021-2022 National Survey on Drug Use and Health: Model-Based Prevalence Estimates (50 States and the District of Columbia). Accessed August 7, 2024. https://www.samhsa.gov/data/report/2021-2022-nsduh-state-prevalence-estimate

© 2025 Stewart MT et al. *JAMA Network Open.*
