# Supplementary material for: Alcohol Use Disorder Medication Coverage and Utilization Management in Medicaid Managed Care Plans
Source: JAMA Netw Open. 2025 Mar 13;8(3):e250695. doi: 10.1001/jamanetworkopen.2025.0695 (PMC11907321; doi:10.1001/jamanetworkopen.2025.0695)
Supplement: Supplement 2. — Data Sharing Statement [file jamanetwopen-e250695-s002.pdf]

Data Sharing Statement

Stewart. Alcohol Use Disorder Medication Coverage and Utilization Management in Medicaid Managed Care Plans. *JAMA Netw Open*. Published March 13, 2025.
doi:10.1001/jamanetworkopen.2025.0695

Data
Data available: Yes
Data types: Data (not involving human participants)
How to access data: stewartm@bu.edu
When available: beginning date: 05-01-2026

Supporting Documents
Document types: None

Additional Information
Who can access the data: researchers whose proposed use of the data has been approved
Types of analyses: for a specified purpose
Mechanisms of data availability: with a signed data access agreement
